# Corneal Sensory Denervation Causes Epithelial Ferroptosis and Delayed Healing in Mice

**DOI:** 10.1167/iovs.66.6.28

**Published:** 2025-06-09

**Authors:** Ning Wang, Yizhou Li, Xiaolei Wang, Lingling Yang, Jing Zhang, Jun Cheng, Xiaoyue Jiang, Xia Qi, Chao Wei, Qingjun Zhou, Ya Li, Suxia Li

**Affiliations:** 1 Eye Institute of Shandong First Medical University, Qingdao Eye Hospital of Shandong First Medical University, Qingdao, China; 2 State Key Laboratory Cultivation Base, Shandong Provincial Key Laboratory of Ophthalmology, Shandong Eye Institute, Shandong First Medical University & Shandong Academy of Medical Sciences, Qingdao, China; 3 Eye Institute of Shandong First Medical University, Eye Hospital of Shandong First Medical University (Shandong Eye Hospital), Jinan, China

**Keywords:** TRPV1, corneal epithelium, ferroptosis, ferrostatin-1, *Trp53*

## Abstract

**Purpose:**

This study aimed to elucidate the role and mechanism of corneal nerves in regulating epithelial cell response against ferroptosis.

**Methods:**

Denervated mouse models were established via surgical axotomy and capsaicin treatment. Monochlorobimane staining was employed to detect cellular glutathione (GSH) levels in the corneal epithelium, and real-time quantitative PCR and immunofluorescence staining were used to evaluate GSH-related gene expression in denervated models and corneas of patients with neurotrophic keratitis. Scanning electron microscopy was utilized to observe mitochondrial morphology in corneal epithelial cells. Ferroptosis inhibitor ferrostatin-1 was administered post-corneal scrape in capsaicin-treated mice, followed by transcriptomic sequencing. The p53 agonist Kevetrin activated p53 in scraped corneas and cultured corneal epithelial cells. Furthermore, capsaicin was topically applied to *Trp53*^+/−^ mice, followed by corneal epithelial scraping.

**Results:**

In denervated models, the expression of GSH-related genes was downregulated, and mitochondrial morphology exhibited characteristics of ferroptosis in corneal epithelial cells. The delay in corneal wound healing induced by TRPV1^+^ sensory denervation was ameliorated by ferrostatin-1 treatment. RNA sequencing and immunofluorescence staining demonstrated upregulated p53 in TRPV1-denervated mice, which was subsequently downregulated following ferrostatin-1 treatment. Kevetrin exacerbated wound healing delays, whereas *Trp53*^+/−^ mice exhibited accelerated healing post-capsaicin denervation compared to wild-type controls.

**Conclusions:**

TRPV1^+^ sensory nerves play a regulatory role in preventing ferroptosis of corneal epithelial cells through the p53/AKT/mTOR signaling pathway. Targeting this pathway may offer therapeutic potential for neurotrophic keratopathy and related disorders.

The corneal nerves play a critical role in maintaining corneal transparency and homeostasis of the corneal epithelium.^1^ Patients with corneal sensory neuropathy may manifest symptoms such as edema, punctate defects, persistent defects, and ulcers attributed to corneal nerve impairment.^2^^,^^3^ Corneal nerve contains three distinct receptor types sensitive to various external stimuli, including multimodal nociceptors, purely mechanical nociceptors, and cold sensory receptors.^4^^,^^5^ Interestingly, the transient receptor potential cation channel, subfamily V member 1 (TRPV1), constitutes approximately 45% of corneal sensory nerves.^6^^,^^7^ TRPV1^+^ neurons actively interact with the immune system by releasing neuropeptides stored within peripheral nerve endings.^8^^–^^10^ In the context of bacterial lung infections, TRPV1 plays a crucial role in regulating pulmonary immunity, and targeting neuroimmune communication through calcitonin gene-related peptide enhances the host defense against pneumonia.^11^ Additionally, TRPV1^+^ nociceptors exhibit a protective effect in mouse intestinal inflammation. Depletion of TRPV1^+^ nociceptor leads to decreased substance P (SP) levels and microbial dysbiosis, and SP protects intestinal tissues in a microbiota-dependent manner.^12^ In cornea, the application of resiniferatoxin or capsaicin, the agonists of TRPV1 receptor, induces the degeneration of corneal TRPV1^+^ sensory nerves, resembling symptoms of corneal sensory neuropathy.^13^^–^^15^ The absence of TRPV1 impairs re-epithelialization by inhibiting the migration and proliferation of epithelial cells.^10^^,^^16^ This effect is regulated by reducing the recruitment of neutrophils and γδT cells, decreasing the number of C-C chemokine receptor 2 (CCR2^+^) macrophages and increasing the number of CCR2 macrophages through receptor activity modifying protein 1 (RAMP1) and somatostatin receptor type 5 (SSTR5) signaling.^10^ These findings suggest that TRPV1^+^ sensory nerves play a critical role in the regulation of corneal epithelial wound healing; however, the precise mechanisms involved still need to be clarified.

Ferroptosis, a regulated form of cell death, is triggered by the accumulation of ferrous ions, resulting in lipid membrane phospholipid peroxidation and the generation of numerous free radicals.^17^ This process culminates in lipid membrane rupture and cell death. Ferroptosis is mechanistically linked to the pathogenesis and progression of diverse diseases, including neurodegenerative disorders (e.g., Alzheimer's, Parkinson's), therapy-resistant cancers, and ischemia–reperfusion-induced organ injury, via iron-mediated lipid peroxidation cascades.^18^^–^^20^ For example, brain tissues from individuals with Alzheimer's disease exhibit abnormalities in iron metabolism and significant lipid peroxidation reactions, both characteristic features of ferroptosis.^21^ Similarly, research has indicated that the reduction in glutathione peroxidase 4 (GPX4) expression and system xc activity in the substantia nigra of Parkinson's disease patients contributes to iron-induced cell death.^22^ Except for the accumulation of ferrous ions, GPX4 plays a pivotal regulatory role in the process of ferroptosis.^23^ Typically, lipid hydroperoxides are reduced to hydrogen derivatives with the involvement of GPX4 and reduced glutathione (GSH). However, when the expression of the GPX4 is diminished or GSH synthesis is compromised, cells become more susceptible to ferroptosis.^24^^,^^25^ In cornea, GSH, synthesized primarily by corneal epithelial cells,^26^ plays a crucial role in maintaining normal redox levels and eliminating exogenous substances such as ultraviolet radiation and particulate matter.^27^^,^^28^ In GPX4^+/−^ mice, there is a significant decrease in the healing rate of the corneal epithelium compared to GPX4^+/+^ mice. Additionally, the application of ferrostatin-1 could reverse corneal epithelial cell death caused by GPX4 deletion.^29^ Despite studies indicating that both TRPV1^+^ nerve degeneration and GPX4 deletion impair corneal wound healing, there is currently no research investigating whether the degeneration of TRPV1^+^ sensory nerve directly influences the expression of GPX4 in corneal epithelial cells.

In this study, surgical denervation and capsaicin-mediated ablation of TRPV1^+^ nerves, as well as patients diagnosed with neurotrophic keratitis, were introduced to assess the association between corneal nerves and ferroptosis of epithelial cells. Furthermore, the therapeutic effects of ferrostatin-1 on corneal wound healing were assessed subsequent to the denervation of TRPV1^+^ sensory nerves. RNA sequence analysis was used to provide insight into the signaling pathway modulated by ferrostatin-1 on the corneal epithelium after TRPV1^+^ sensory denervation. Moreover, *Trp53*^+/−^ mice were used along with the p53 agonist Kevetrin to elucidate the downstream pathway. These findings illuminate the pivotal role of TRPV1^+^ sensory nerves in maintaining corneal epithelial homeostasis and underscore the potential of ferrostatin-1 as a promising treatment for diseases associated with corneal nerve denervation.

## Materials and Methods

### Animal Models

Adult males C57BL/6J mice (8 weeks old) purchased from the Charles River Laboratory (Beijing, China). *Trp53*^+/−^ mice were purchased from Shanghai Model Organisms Center, Inc. (NM-KO-00011; Shanghai, China). The primers used for genotyping of *Trp53*^+/−^ mice and identification results are shown in Supplementary Table S1 and Supplementary Figure S1, respectively. All animals were treated according to the ARVO Statement for the Use of Animals in Ophthalmic and Vision Research.

All mice were intraperitoneally injected with 50 mg/kg pentobarbital sodium for general anesthesia and topically anesthetized with 2% xylocaine. The corneal sensory neuropathy model was established as described in a previous study,^30^ wherein forceps were used to compress the nerve bundle for 45 seconds. Ofloxacin eye ointment was applied to avoid infection. After 24 hours of surgery, corneal epithelial defects were observed by staining with 0.25% sodium fluorescein and documented using a slit-lamp microscope (BQ900; Haag-Streit, Köniz, Switzerland).

For the pharmacological denervated model, C57BL/6J mice were anesthetized as described above and then topically treated with 1-mM capsaicin or solvent (HY-10448; MedChemExpress, Monmouth Junction, NJ, USA) for 20 minutes. This procedure was repeated for two consecutive days. On the third day, photographs were taken after staining with 0.25% sodium fluorescein.

### Human Corneal Tissues

Human corneal tissues were obtained from patients with neurotrophic keratitis, and normal corneal tissues were obtained from healthy individuals through donation. The detailed information is provided in Supplementary Table S2. The study was conducted in accordance with the tenets of the Declaration of Helsinki and approved by the Medical Ethics Committee of Qingdao Eye Hospital, Shandong First Medical University.

### Corneal Epithelial Debridement

Mice were intraperitoneally injected with pentobarbital sodium and topically anesthetized with xylocaine. The corneal epithelium, marked with a 2-mm trephine, was gently scraped using the Algerbrush II Rust Ring Remover (The Alger Company, Mansfield, MA, USA). Subsequently, a subconjunctival injection of 1-mM capsaicin was administered, and ofloxacin eye ointment was applied to prevent infection. The healing progress of the corneal epithelium was observed by staining with 0.25% fluorescein sodium and was photographed using a slit lamp at 0, 12, and 36 hours after the corneal abrasion. Additionally, ferrostatin-1 (HY-100579, 5 µL, 220 µM; MedChemExpress) dissolved in 0.1% dimethyl sulfoxide (DMSO) or solvent was topically applied (four times a day) for 2 days after corneal epithelial wounding.

In C57BL/6J mice and *Trp53*^+/−^ mice, the corneal epithelium, marked with a 2.5-mm trephine, was gently scraped. Concurrently, *Trp53*⁺^/^⁻ mice and wild-type mice underwent subconjunctival administration of 1-mM capsaicin solution, followed by prophylactic application of ofloxacin ophthalmic ointment to mitigate potential bacterial infection. Kevetrin (HY-16271; MedChemExpress) was administered topically to C57BL/6J mice at a dosage of 5 µL (1.8 µg/µL) four times a day, with equivalent volumes of vehicle control applied following the same regimen. The corneal wound healing of C57BL/6J and *Trp53*^+/−^ mice was assessed by staining with 0.25% fluorescein sodium and documented using a slit lamp at 0, 24, and 36 hours after the corneal abrasion. The defect area was analyzed using ImageJ (National Institutes of Health, Bethesda, MD, USA).

### Corneal Sensitivity

The Cochet–Bonnet esthesiometer (Luneau Ophtalmologie, Chartres Cedex, France) was utilized to measure corneal sensitivity. The cornea was gently touched with a nylon wire of maximum length, which was then gradually shortened by 0.1-cm increments until a blink reaction was observed. The length of the nylon filament at which the first blink reaction occurred was recorded as the threshold for corneal sensitivity. This process was repeated three times to ensure accuracy.

### Measurement of Intracellular GSH Level

Intracellular GSH levels were measured using monochlorobimane (dissolved in DMSO; Sigma-Aldrich, St. Louis, MO, USA). Frozen sections (7 µm) of eyeball were incubated with monochlorobimane (50 µM) at 37°C for 30 minutes, and images were captured immediately with a Revolve microscope (Echo Laboratories, San Diego, CA, USA).

### Mitochondria Evaluation

The corneal tissue was promptly fixed at 4°C for 2 to 4 hours using an electron microscope fixative (B0012, 2.5% glutaraldehyde; Aidisheng Biotechnology, Jiangsu, China). The use of graded ethanol solutions for dehydration and acetone and 812 embedding agent for infiltration further ensured minimal disruption to cellular structures. Fixed corneal epithelial samples were embedded in a plastic resin and sectioned using an ultramicrotome equipped with a diamond knife, as is standard practice for biological samples. This method ensures uniform sample thickness, typically around 60 to 80 nm, which is optimal for transmission electron microscopy (TEM) imaging and minimizes the risk of structural distortion. TEM was used to observe the mitochondrial morphology of the sample at an accelerating voltage of 100 kV (JEM-1400; JEOL, Tokyo, Japan).

### Corneal Epithelial Cell Culture and Treatment

Human corneal epithelial cells (HCECs) were kindly provided by Choun-Ki Joo (The Catholic University of Korea, Seoul, Korea). HCECs were cultured at 37°C in Dulbecco's Modified Eagle Medium/Nutrient Mixture F-12 (DMEM/F-12; Sigma Aldrich) containing 10% Gibco fetal bovine serum (Thermo Fisher Scientific, Waltham, MA), 100 U/mL penicillin, and 100 µg/mL Gibco streptomycin.

HCECs were seeded in 96-well plates at a density of 5000 cells per well and treated with 160-µM Kevetrin for 48 hours. Following that, proteins were extracted and analyzed by western blot. Cell proliferation rates were measured using the Cell Counting Kit-8 (CCK-8) colorimetric assay (Beyotime, Shanghai, China). For the cell migration assay, HCECs were plated on 12-well plate until confluence. After they were starved overnight, a wound was created using a micropipette tip, and the cells were then incubated with or without Kevetrin (160 µM) for 24 hours. Quantitative assessment of migration was determined by measuring the wound closure of photographs using ImageJ.

### Immunofluorescence Staining

For whole-mount staining, mouse corneas were fixed in 4% paraformaldehyde (Biosharp Life Sciences, Guangzhou, China) for 1 hour on ice and then permeabilized and blocked in PBS containing 0.3% Triton X-100 solution (Solarbio, Beijing, China) and 3% bovine serum albumin (Solarbio) overnight at 4°C. Subsequently, the corneas were incubated with Alexa Fluor 647 anti-Tubulin Beta 3 (TUBB3) Antibody (657406; BioLegend, San Diego, CA, USA) at 4°C for 12 hours. Finally, the tissues were observed and captured using a ZEISS LSM880 inverted microscope (Carl Zeiss Microscopy, Oberkochen, Germany).

For immunostaining, mouse eyeballs and human corneal tissues were embedded in Tissue-Tek OCT compound (Sakura Finetek, Tokyo, Japan) at −80°C, and 7-µm frozen corneal sections were prepared. These frozen corneal sections were fixed with 4% paraformaldehyde for 15 minutes and permeabilized with 0.1% Triton X-100 for 30 minutes, followed by blocking with a 5% bovine serum albumin solution for 2 hours at room temperature. Then, the sections were incubated with GPX4 antibodies (DF6701; Affinity Biosciences, Changzhou, China), GSTM1 antibodies (12412-1-AP; Proteintech, Rosemont, IL, USA), p53 antibodies (10442-1-AP; Proteintech), p-AKT antibody (4060; Cell Signaling Technology, Danvers, MA, USA), or p-mTOR antibodies (ab109268; Abcam, Cambridge, UK) overnight at 4°C. Following this, the sections were incubated with Invitrogen Goat anti-Rabbit IgG (H+L) Cross-Adsorbed Secondary Antibody, Alexa Fluor 488 (A-11008; Thermo Fisher Scientific) for 1 hour at room temperature. After staining with 4′,6-diamidino-2-phenylindole (DAPI; Solarbio) for 5 minutes, the sections were observed and captured using the Echo Laboratories Revolve microscope and ZEISS LSM 880 inverted microscope.

### Real-Time Quantitative PCR Reaction

The corneal epithelium was collected and total RNA was extracted using the TransZol Up Plus RNA Kit (TransGen, Beijing, China) and subsequently reverse transcribed into cDNA using the HiScript III RT SuperMix (VaZyme, Shanghai, China). Gene expression levels were analyzed using ChamQ Universal SYBR qPCR Master Mix (VaZyme) with the primers listed in Supplementary Table S3 by using the 7500 Real-Time PCR System (Applied Biosystems, Foster City, CA, USA). The specific procedure was as follows: Pre-denaturation at 95°C for 30 seconds was followed by 40 cycles of reactions divided into two steps (10 seconds at 95°C and 30 seconds at 60°C). Data analysis was performed using the 2^−ΔΔCt^ method, with β-actin used as the internal reference.

### RNA Sequencing and Bioinformatics Analysis

The corneal epithelium was individually collected from the control, capsaicin-treated, and capsaicin-treated and supplemented with ferrostatin-1 groups and stored in liquid nitrogen (24 hours). Transcriptome sequencing and analysis were performed by OE Biotech Co., Ltd. (Shanghai, China). Differential expression analysis was conducted using the DESeq (2012) R package. To visualize the expression pattern of genes in different groups and samples, hierarchical cluster analysis of the differentially expressed genes (DEGs) was performed. Additionally, Kyoto Encyclopedia of Genes and Genomes (KEGG) pathway enrichment analyses of DEGs was carried out using R, based on the hypergeometric distribution. The online platform provided by OE Biotech was used for Gene Set Enrichment Analysis (GSEA) and to draw volcano plots. |Log_2_(FC)| > 1.5 and *P* < 0.05 were defined as significant differences.

### Western Blot Analysis

The total protein (30 µg) was separated by 6% or 12% SDS-PAGE and transferred onto a methanol-pretreated polyvinylidene fluoride membrane (Millipore, Billerica, MA, USA). The bolts were blocked with 5% nonfat dry milk for 2 hours at room temperature and then incubated overnight at 4°C with specific antibodies: p53, p-AKT, AKT (A17909; Affinity Biosciences), p-mTOR, mTOR (ab134903; Abcam), and β-actin (66009-1; Proteintech). Following primary antibody incubation, blots were washed three times and subsequently incubated with a horseradish peroxidase–conjugated secondary antibody (Proteintech) for 2 hours. The blots were visualized using a chemiluminescence detection kit (Millipore).

### Statistical Analysis

The statistical analysis was performed using Prism 9.0 (GraphPad Software, Boston, MA, USA) and analyzed with Student's *t*-test or ANOVA test. A significance threshold of *P* < 0.05 was adopted to determine statistical significance.

## Results

### Nerve Degeneration Triggered Ferroptosis of Corneal Epithelial Cells

**Figure 1.**
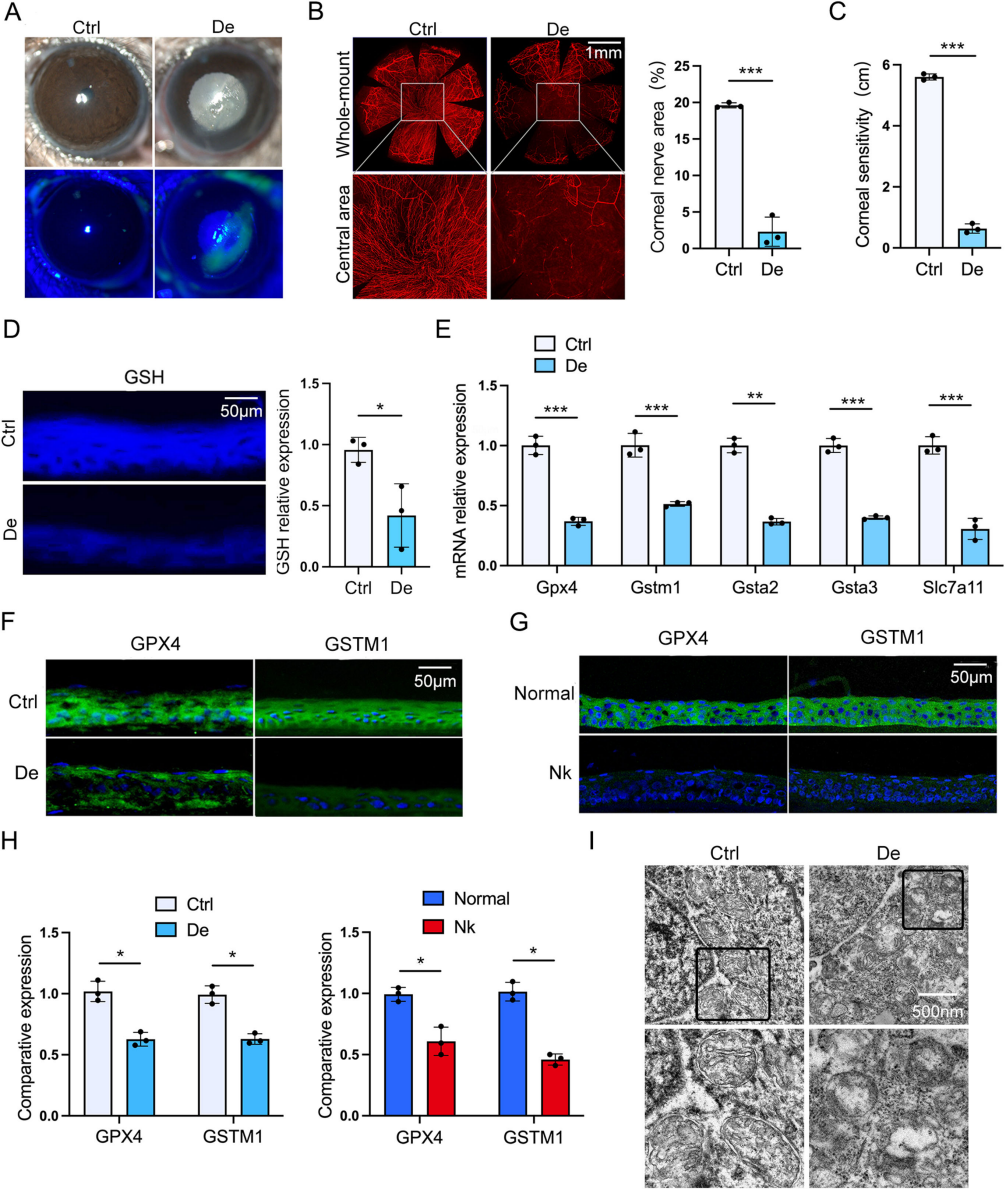
Nerve degeneration induces the ferroptosis of corneal epithelial cells. (**A**) The corneal epithelial defect of surgical denervated mice was observed with slit-lamp and fluorescein sodium staining. (**B**) Corneal nerve degeneration was detected by whole-mount βIII-tubulin staining, and the percentage of nerve-covered area was analyzed by ImageJ. (**C**) Corneal sensation was measured using a Cochet–Bonnet esthesiometer. (**D**) Intracellular GSH was detected by monochlorobimane staining and analyzed by ImageJ. (**E**) The levels of *Gpx4*, *Gstm1*, *Gsta2*, *Gsta3*, and *Slc7a11* were analyzed by quantitative PCR. (**F**–**H**) The results of immunofluorescence in the expression of GPX4 and GSTM1 proteins in the corneal epithelium of the denervated mice. The expression of GPX4 and GSTM1 in the corneal epithelium of surgical corneal denervated mice (F) and patients with neurotrophic keratitis (**G**) was detected by immunofluorescence staining, and the fluorescence intensity was quantified by ImageJ (**H**). (**I**) The mitochondrial morphology of corneal epithelium was observed by transmission electron microscopy after surgical corneal denervation. Data are presented as mean ± SEM. *P < 0.05, **P < 0.01, ***P < 0.001.

To determine the specific mode of corneal epithelial cell death associated with denervation, we established a model of corneal denervation by squeezing the ciliary long and short nerve bundles for 45 seconds using forceps bundles.^30^ At 24 hours postoperatively, the corneal denervated mice showed the spontaneous corneal epithelial detachment (Fig. 1A) and regression of corneal nerves (Fig. 1B), accompanied with decreased corneal sensitivity of 0.6 ± 0.1 cm compared to the sham-operated mice (5.6 ± 0.1 cm) (Fig. 1C). Moreover, the monochlorobimane staining showed that GSH levels in corneal epithelium decreased approximately 2.3-fold after corneal denervation compared to the sham-operated mice (Fig. 1D). To further confirm the roles of TRPV1+ sensory nerves in regulating corneal epithelial GSH metabolism, we detected the key genes involved in GSH metabolism. *Gpx4* encodes glutathione peroxidase 4, which is essential for scavenging lipid hydroperoxides and preventing oxidative damage to cell membranes. *Gstm1*, a member of the glutathione *S*-transferase family, interacts with GPX4 and modulates its expression, thereby influencing ferroptosis susceptibility. *Gsta2* and *Gsta3* are also involved in GSH metabolism, contributing to the synthesis and recycling of GSH, which is a key antioxidant in cells. The expression of these genes can scavenge peroxides and reactive oxygen species (ROS) in cells, thereby protecting them from oxidative stress-induced damage.^31^^,^^32^ The results showed that the mRNA levels of *Gpx4*, *Gstm1*, *Gsta2*, *Gsta3*, and *Slc7a11* were downregulated in the corneal epithelium of corneal denervated mice (Fig. 1E). Moreover, both GPX4 and GSTM1 were significantly reduced in corneal epithelium of denervated mice (Figs. 1F, 1H). Similarly, the decreased GPX4 and GSTM1 expression was also found in patients with neurotrophic keratitis (Figs. 1G, 1H). Importantly, GPX4 also plays a critical role in preventing ferroptosis by inhibiting the iron-dependent accumulation of lipid hydroperoxides.^33^ To investigate whether ferroptosis occurred in corneal epithelial cells after nerve ablation, the mitochondrial morphology of corneal epithelial cells was detected by scanning electron microscopy. The results showed the mitochondrial changes indicative of ferroptosis in corneal epithelial cells of denervated mice (Fig. 1I).

### TRPV1^+^ Sensory Degeneration Induced Ferroptosis in Corneal Epithelial Cells

**Figure 2.**
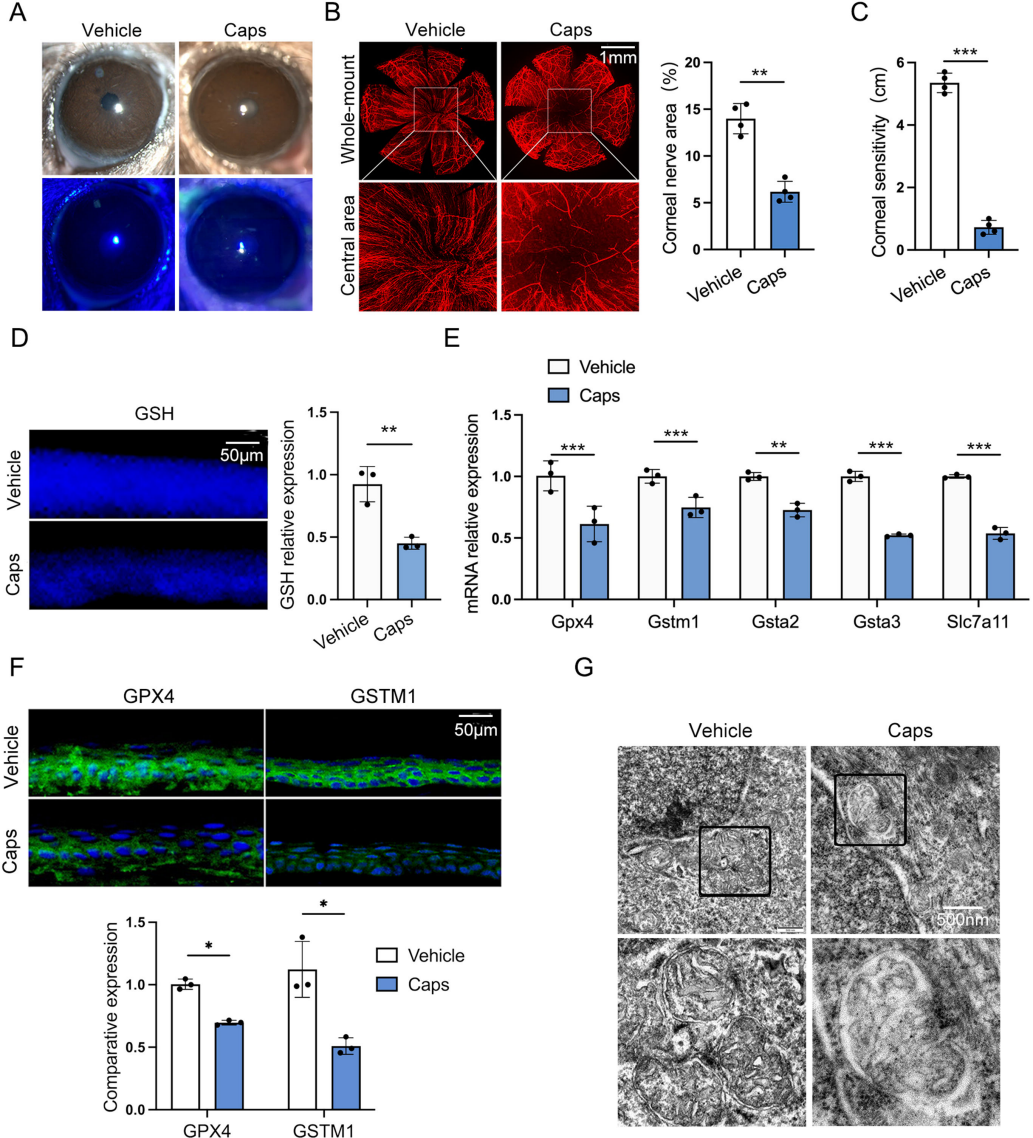
TRPV1^+^ sensory degeneration triggers the ferroptosis of corneal epithelial cells. (**A**) The ocular surface of capsaicin (Caps)-treated mice was observed with slit-lamp and fluorescein sodium staining. (**B**) Corneal nerve degeneration was detected by βIII-tubulin staining and the percentage of nerve-covered area was analyzed by ImageJ. (**C**) Corneal sensation was measured using a Cochet–Bonnet esthesiometer. (**D**) Intracellular GSH was detected by monochlorobimane staining and analyzed by ImageJ. (**E**) The levels of *Gpx4*, *Gstm1*, *Gsta2*, *Gsta3*, and *Slc7a11* were analyzed by quantitative PCR. (**F**) The expression of GPX4 and GSTM1 in the corneal epithelium of capsaicin-treated mice was detected by immunofluorescence staining and quantified by ImageJ. (**G**) The mitochondrial morphology of corneal epithelium was observed by transmission electron microscopy after capsaicin treatment. Data are presented as mean ± SEM. *P < 0.05, **P < 0.01, ***P < 0.001.

TRPV1^+^ sensory nerve constitutes approximately 45% of corneal sensory nerves.^4^^,^^5^ To assess whether TRPV1^+^ sensory denervation induced ferroptosis of corneal epithelial cells, the capsaicin was utilized to establish a corneal TRPV1^+^ sensory-denervated model. The ocular surface remained intact after being topically treated with 1-mM capsaicin (Fig. 2A). However, regression of corneal nerves (Fig. 2B) and decreased corneal sensitivity (Fig. 2C) were found in capsaicin-treated mice. Consistent with the results of surgical corneal denervated mice, the corneal epithelial GSH levels, as well as *Gpx4*, *Gstm1*, *Gsta2*, *Gsta3*, and *Slc7a11* levels were downregulated in the corneal epithelium of capsaicin-treated mice (Figs. 2D–2F). To investigate whether ferroptosis occurred in corneal epithelial cells after TRPV1^+^ sensory nerve ablation, the mitochondrial morphology of corneal epithelial cells was detected by scanning electron microscopy. The results showed that the mitochondrial morphology of corneal epithelial cells exhibited features of ferroptosis after capsaicin treatment (Fig. 2G). The results indicated that the ferroptosis occurred in corneal epithelial cells after TRPV1^+^ sensory denervation, although spontaneous corneal epithelial detachment had not been observed.

### Ferrostatin-1 Reversed the Delay of Corneal Wound Healing in TRPV1^+^ Sensory-Denervated Mice

**Figure 3.**
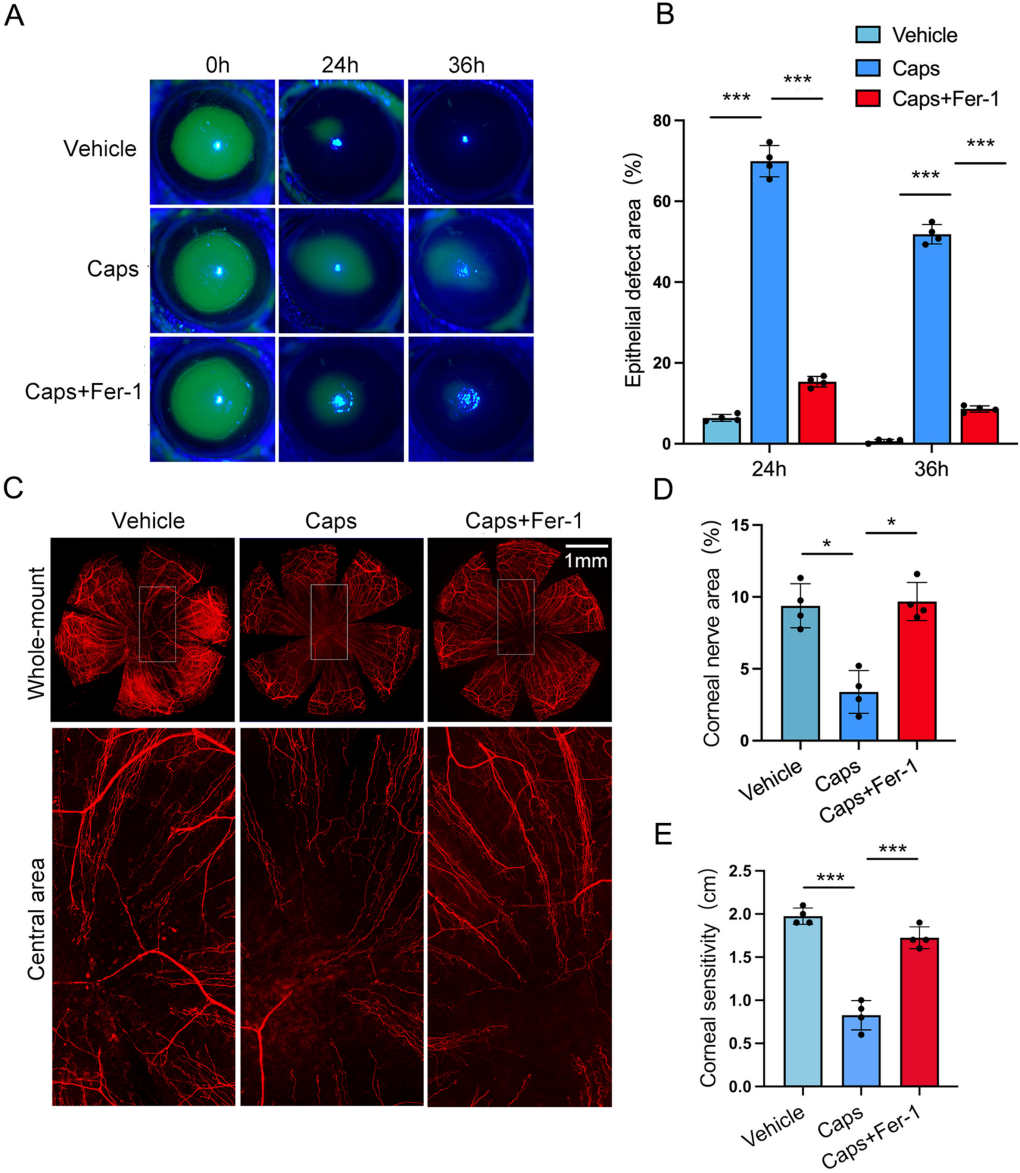
Ferrostatin-1 reverses the delay of corneal wound healing caused by capsaicin. (**A**) The corneal epithelium of mice treated with vehicle, capsaicin, or capsaicin plus ferrostatin-1 (Fer-1) was stained with fluorescein sodium at 0, 24, and 36 hours after scraping. (**B**) Histogram of residual epithelial defect was presented as the percentage of the original wound. (**C**) Corneal nerve regeneration of vehicle, capsaicin, and capsaicin plus ferrostatin-1 was detected with βIII-tubulin staining at 48 hours after scraping. (**D**) The percentage of nerve-covered area. (**E**) Corneal sensation was measured at 48 hours after scraping. Data are presented as mean ± SEM. *P < 0.05, **P < 0.01, ***P < 0.001.

TRPV1^+^ sensory denervation leads to the delay of wound healing in cornea and skin.^15^^,^^34^^,^^35^ To investigate whether ferroptosis of corneal epithelium plays an important role in maintaining corneal homeostasis, ferrostatin-1 was utilized in capsaicin-treated mice during corneal wound healing. The results showed that topical ferrostatin-1 application significantly promoted corneal epithelial healing in capsaicin-treated mice (Figs. 3A, 3B). Moreover, whole-mount βIII-tubulin staining showed that the corneal nerve regeneration significantly improved in the capsaicin-treated mice after ferrostatin-1 application (Figs. 3C, 3D), accompanied with improved corneal sensitivity of 1.7 ± 0.1 cm compared to the capsaicin-treated mice (0.8 ± 0.1 cm) (Fig. 3E). These results indicate that ferrostatin-1 reversed the delay of wound healing caused by TRPV1^+^ sensory denervation.

### Ferrostatin-1 Decreased p53 Levels in Corneal Epithelial Cells After TRPV1^+^ Sensory Denervation

**Figure 4.**
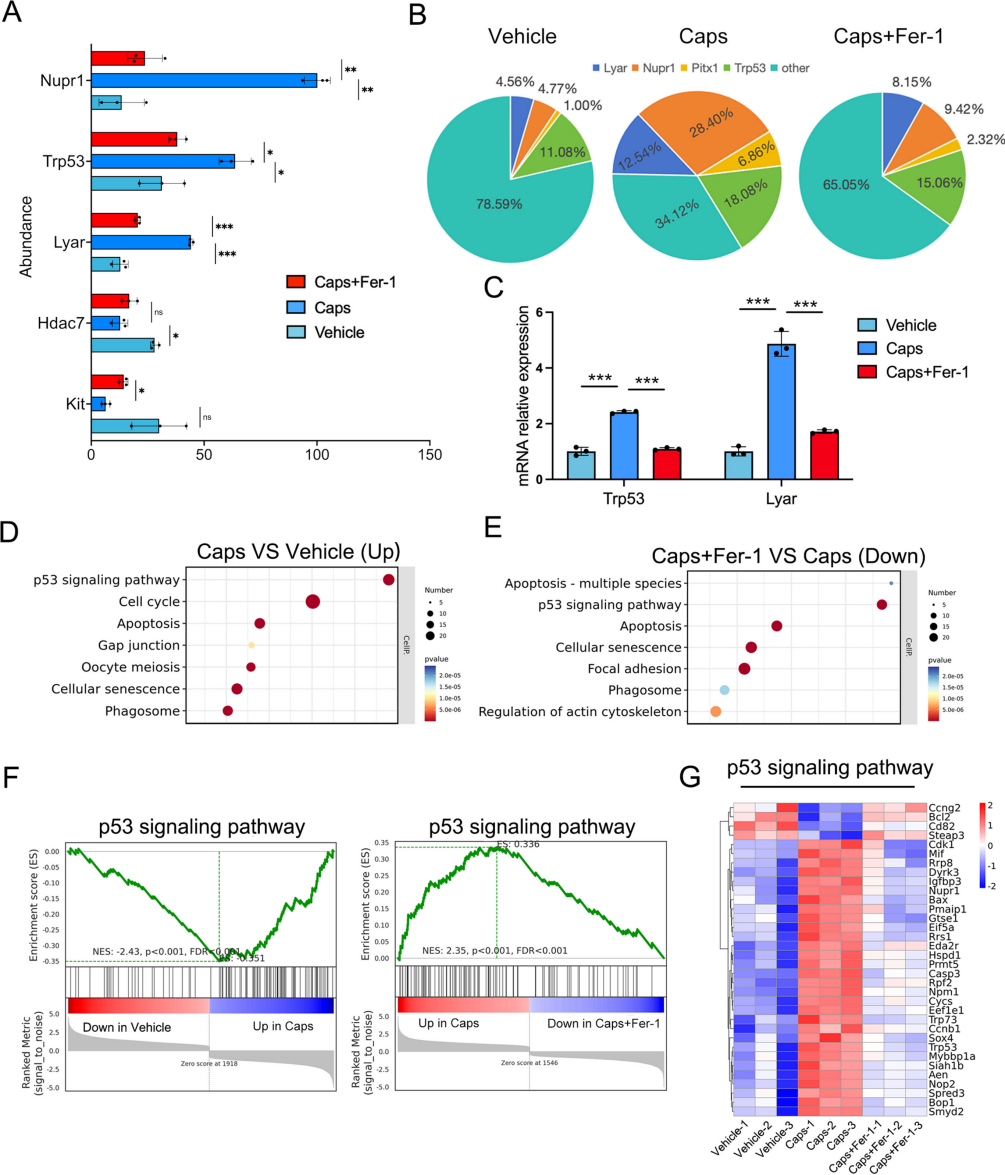
Ferrostatin-1 downregulates the p53 signaling axis elevated in the corneal epithelium of capsaicin-treated mice. (**A**) Relative expression of the top five transcription factors in capsaicin-treated mice with or without ferrostatin-1 application and the vehicle group. (**B**) The proportion of changes of transcription factors within the total pool of transcription factors analyzed in capsaicin-treated mice with or without ferrostatin-1 application and the vehicle group. (**C**) The levels of *Trp53* and *Lyar* in the corneal epithelium of mice treated with vehicle, capsaicin, or capsaicin plus ferrostatin-1 were analyzed by quantitative PCR. (**D**) KEGG analysis of upregulated signaling pathways related to cellular processes in the corneal epithelium of capsaicin-treated mice compared with vehicle group. (**E**) KEGG analysis of downregulated signaling pathways related to cellular processes) after ferrostatin-1 application in capsaicin-treated mice. (**F**) Enrichment profiles of the p53 signaling pathway analyzed by GSEA. (**G**) Heatmap of genes involved in p53 signaling pathway.

To further confirm the mechanism of ferroptosis of corneal epithelial cells after corneal TRPV1^+^ sensory denervation, corneal epithelium was collected for RNA sequencing and bioinformatic analyses. The principal component analysis graphs showed the biological replication of each group (Supplementary Fig. S2A), and Venn diagrams indicated that 1453 genes exhibited changes after ferrostatin-1 application in capsaicin-treated mice (Supplementary Fig. S2B). Among them, 811 genes were jointly upregulated, and 641 genes were jointly downregulated (Supplementary Fig. S2C). As shown in Figure 4A, the transcription factors *Nupr1* and *Trp53* were elevated in TRPV1^+^ nerve-degenerated mice, but they decreased significantly after ferrostatin-1 application (Figs. 4A, 4B).

Nuclear protein 1 (NUPR1) expression is upregulated during ferroptosis induced by RAS-selective lethal 3 (RSL3) and erastin, and this upregulation is mediated by activating transcription factor 4 (ATF4). However, NUPR1 has been shown to inhibit iron-dependent oxidative damage during ferroptosis and acts as a repressor of ferroptosis.^36^^,^^37^ The elevated NUPR1 levels observed after TRPV1^+^ sensory denervation may represent an adaptive response of cells to cope with oxidative stress and maintain cellular homeostasis. Moreover, Ly1 antibody reactive clone (LYAR) was significantly upregulated in TRPV1-denervated mice, but its expression was markedly reduced following ferrostatin-1 treatment (Figs. 4A, 4B). LYAR has been previously reported to promote cell-cycle progression, and its knockdown has been shown to inhibit cell proliferation and induce G0/G1 cell-cycle arrest.^38^ These findings suggest that LYAR may contribute to the observed cell-cycle upregulation in TRPV1-denervated mice. The receptor tyrosine kinase KIT plays critical roles in mediating cellular differentiation, proliferation, and survival.^39^ The expression of KIT was downregulated in TRPV1-denervated mice (Fig. 4A), raising the possibility that KIT signaling might influence the fate of epithelial cells in this context. Furthermore, KEGG pathway enrichment analysis revealed a notable upregulation of the p53 signaling pathway following TRPV1^+^ sensory denervation (Fig. 4C). Conversely, the p53 signaling pathway was significantly downregulated in TRPV1^+^ sensory-denervated mice after treatment with ferrostatin-1 (Fig. 4D). The GSEA plot indicated that most of the genes in the p53 signaling pathway were upregulated following TRPV1^+^ sensory denervation, but they were downregulated after ferrostatin-1 application (Fig. 4E). Moreover, heatmaps displayed the distinctive gene expression patterns associated with the p53 signaling pathway (Fig. 4F).

**Figure 5.**
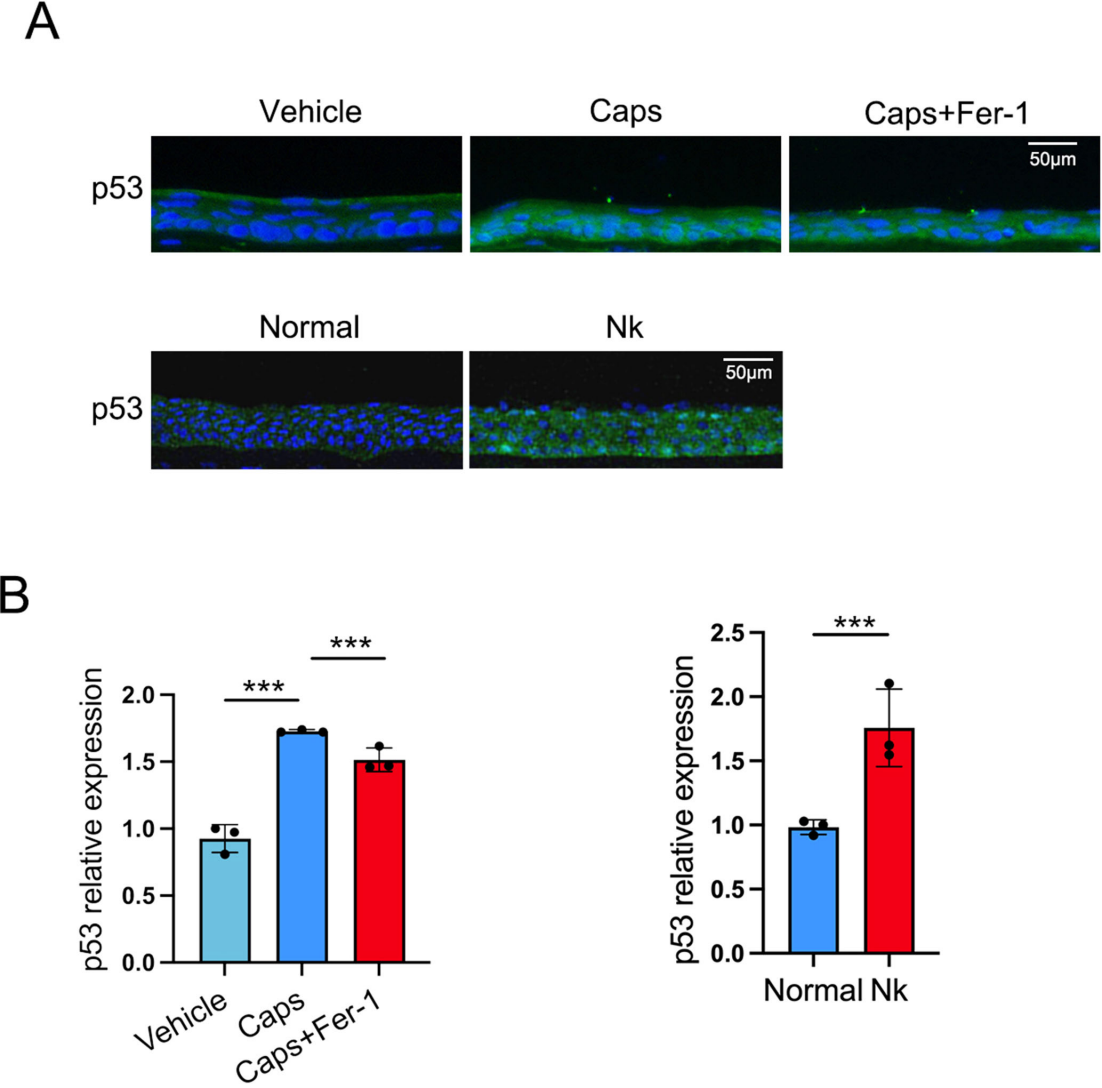
Ferrostatin-1 reduces p53 level in the corneal epithelium of capsaicin-treated mice. (**A**, **B**) The expression of p53 in the corneal epithelium of mice treated with vehicle, capsaicin, or capsaicin plus ferrostatin-1 and in patients with neurotrophic keratitis was detected by immunofluorescence staining (**A**) and quantified by ImageJ. (**B**). Data are presented as mean ± SEM. **P < 0.01, ***P < 0.001.

Based on the results of transcriptomics analysis, the levels of *Trp53* in corneal epithelium were measured by quantitative PCR. *Trp53* was upregulated in TRPV1^+^ sensory-denervated mice but was downregulated after ferrostatin-1 application (Fig. 4B). Importantly, increased levels of p53 in corneal epithelium were also detected in TRPV1^+^ sensory-denervated mice and patients with neurotrophic keratopathy (Figs. 5A, 5B). However, the levels of p53 decreased in the corneal epithelium after ferrostatin-1 was applied (Figs. 5A, 5B). Thus, ferrostatin-1 could reverse the elevation of p53 in corneal epithelial cells after TRPV1^+^ sensory denervation.

### Ferrostatin-1 Reversed the p53-Mediated AKT and mTOR Inhibition in Corneal Epithelium of TRPV1^+^ Sensory-Denervated Mice

**Figure 6.**
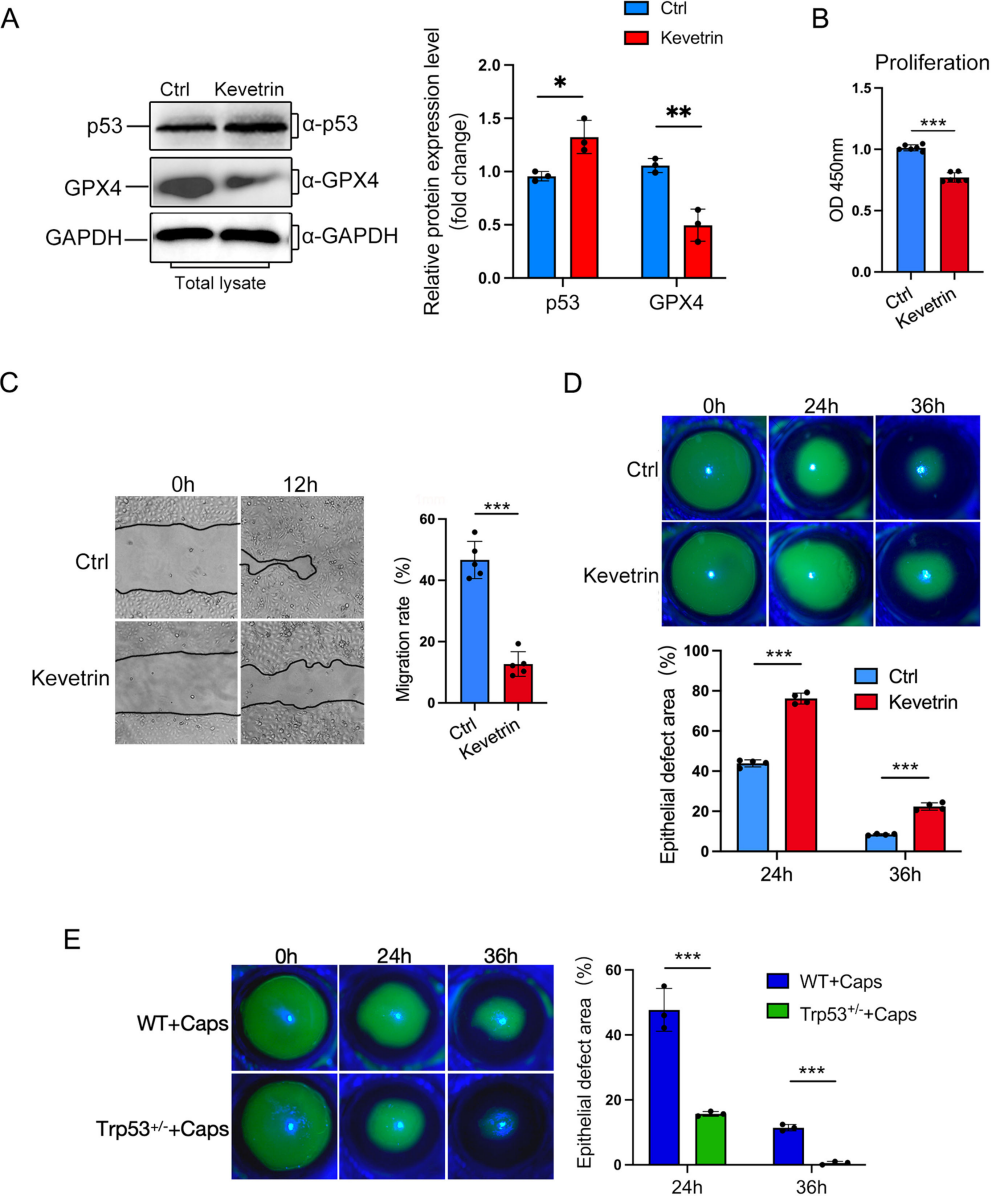
p53 downregulates GPX4 expression and modulates corneal wound healing. (**A**) The expression of p53 and GPX4 in HCECs treated with Kevetrin was determined by western blotting. (**B**) The cell viability of HCECs treated with Kevetrin was determined by CCK-8 assay. (**C**) The migration of HCECs treated with Kevetrin was observed by photography. The migration area was analyzed by ImageJ. (D) The corneal epithelium of Kevetrin-treated and control mice was stained with fluorescein sodium at 0, 24, and 36 hours after scraping (*upper panel*); a histogram of residual epithelial defect shows the percentage of the original wound (*lower panel*). (**E**) The corneal epithelium of *Trp53*^+/−^ and WT mice was stained with fluorescein sodium at 0, 24, and 36 hours after scraping (*upper panel*); a histogram of residual epithelial defect shows the percentage of the original wound (*lower panel*). Data are presented as mean ± SEM. *P < 0.05, **P < 0.01, ***P < 0.001.

To clarify the roles of p53 in regulating corneal epithelial cells, Kevetrin was employed to activate the p53 expression in HCECs. The western blot results showed that the activation of p53 in HCECs resulted in a decrease in GPX4 levels (Fig. 6A). Moreover, the p53 activation inhibited the proliferation and migration of HCECs (Figs. 6B, 6C). To further confirm the specific roles of p53 after TRPV1^+^ sensory nerve denervation, Kevetrin and *Trp53*^+/−^ mice were used in this study. The results revealed that Kevetrin significantly inhibited corneal epithelial wound healing (Fig. 6D). Furthermore, Kevetrin treatment resulted in impaired regeneration of corneal nerve fibers (Supplementary Figs. S3A, S3B) concomitant with a marked reduction in corneal sensitivity (Supplementary Fig. S3C). After TRPV1^+^ sensory nerve denervation, *Trp53*^+/−^ mice exhibited accelerated healing compared to wild-type controls (Fig. 6E).

**Figure 7.**
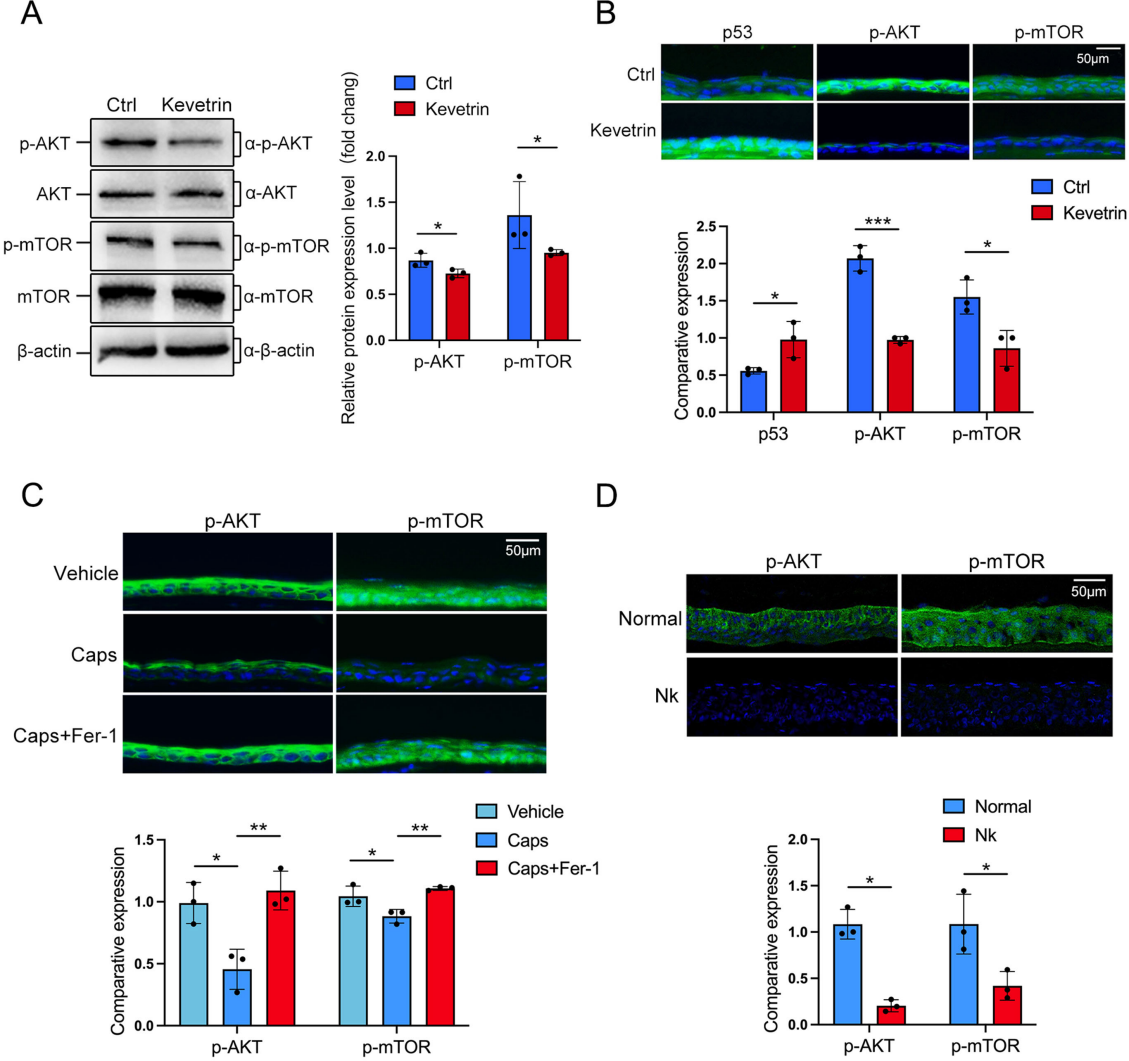
Ferrostatin-1 abolishes p53-mediated AKT and mTOR inhibition in TRPV1^+^ sensory-denervated mice. (**A**) The phosphorylation of AKT and mTOR in HCECs treated with Kevetrin was determined by western blotting. (**B**) The expression of p53, p-AKT, and p-mTOR in the corneal epithelium of Kevetrin-treated mice was detected by immunofluorescence staining, and the fluorescence intensity was quantified by ImageJ. (**C**) The levels of p-AKT and p-mTOR in the corneal epithelium of mice treated with vehicle, capsaicin, or capsaicin plus ferrostatin-1 were detected by immunofluorescence staining (*upper panel*) and quantified by ImageJ (*lower panel*). (**D**) The levels of p-AKT and p-mTOR in the corneal epithelium of patients with neurotrophic keratitis were determined by immunofluorescence staining (*upper panel*) and quantified by ImageJ (*lower panel*). Data are presented as mean ± SEM. *P < 0.05, **P < 0.01, ***P < 0.001.

The AKT/mTOR pathway is crucial for signal transduction and biological regulation, with AKT playing a key role in various cellular activities such as growth, proliferation, cell cycle, and metabolism by activating mTOR.^40^ Moreover, the AKT/mTOR pathway is also involved in regulating the acquisition of extracellular iron ions by cells.^41^ To elucidate the mechanisms underlying the effects of p53 activation after TRPV1^+^ sensory denervation, the phosphorylation levels of AKT and mTOR were detected in p53-activated HCECs. The results showed that the phosphorylation levels of AKT and mTOR significantly decreased with the activation of p53 (Fig. 7A). Similarly, the phosphorylation levels of AKT and mTOR also decreased in the corneal epithelium of Kevetrin-treated mice (Fig. 7B).

To determine whether ferrostatin-1 ameliorated the delay of corneal wound healing after TRPV1^+^ sensory denervation by mediating the p53 signaling pathway, we conducted further investigations into both TRPV1^+^ sensory-denervated mice and patients with neurotrophic keratitis. The downregulation of phosphorylation levels of AKT and mTOR was observed in the corneal epithelium of TRPV1^+^ sensory-denervated mice (Fig. 7C) and patients with neurotrophic keratitis (Fig. 7D). Importantly, phosphorylation levels of AKT and mTOR were elevated after TRPV1^+^ sensory nerve denervation was treated by ferrostatin-1 (Fig. 7C). These results indicate that ferrostatin-1 reversed the delayed wound healing in TRPV1^+^ sensory-denervated mice by abolishing the p53-mediated AKT and mTOR inhibition.

## Discussion

In this study, we observed a decrease in the expression of specific GSH-related genes, such as *Gpx4* and *Gstm1*, and we noted distinctive mitochondrial characteristics indicative of ferroptosis in the corneal epithelial cells after corneal nerve degeneration. However, ferrostatin-1 treatment significantly accelerated corneal wound healing in TRPV1^+^ sensory denervation. KEGG pathway analysis revealed upregulation of the p53 signaling pathway in TRPV1^+^-denervated mice, whereas it was subsequently downregulated after ferrostatin-1 treatment, as confirmed by immunofluorescence staining of p53. Moreover, corneal wound healing was accelerated in *Trp53*^+/−^ mice but delayed in Kevetrin-treated mice due to inhibition of the proliferation and migration of corneal epithelial cells. In TRPV1^+^ sensory-denervated mice, ferrostatin-1 application reduced p53 expression and reactivated the AKT/mTOR pathway of corneal epithelial cells. Collectively, our findings revealed the mechanistic role of ferrostatin-1 in preventing the ferroptosis of corneal epithelium through p53-mediated AKT and mTOR inhibition after TRPV1^+^ sensory denervation.

As a tumor suppressor, p53 plays a crucial role in cell-cycle regulation and DNA damage repair.^42^^,^^43^ Meanwhile, a growing body of research has demonstrated a close connection between p53 expression and ferroptosis. p53 bidirectionally regulates the occurrence of ferroptosis through both canonical and noncanonical pathways according to whether it depends on GPX4. A classic pathway involves p53 inhibiting SLC7A11 expression to reduce GPX4 activity, thereby inducing ferroptosis.^19^ We observed that high concentrations of capsaicin can cause degeneration of TRPV1^+^ sensory nerves in the cornea and delay epithelial wound healing. Concurrently, we discovered that elevated p53 levels and decreased GPX4 levels trigger ferroptosis in corneal epithelial cells, thus representing a critical mechanism underlying the delayed epithelial repair caused by corneal nerve damage. The corneal epithelium receives essential trophic support from sensory nerves originating in the ophthalmic branch of the trigeminal nerve, which form a dense subbasal neural plexus. Denervation disrupts this neurotrophic signaling, leading to diminished levels of neurotrophic factors and neurotransmitters critical for epithelial homeostasis.^44^^,^^45^ Mitochondria, pivotal regulators of energy metabolism, calcium buffering, and ROS scavenging, become dysregulated following denervation. In neurotrophic keratopathy, dysfunctional mitochondria generate excessive ROS and reduced intracellular GSH, the primary ROS scavenger.^46^ Notably, topical administration of SP in diabetic mice reverses this imbalance by suppressing ROS accumulation and restoring GSH levels,^47^ whereas brain-derived neurotrophic factor (BDNF) has been shown to enhance GSH biosynthesis in oocytes.^48^ Collectively, these findings strongly suggest that corneal denervation reduces epithelial GSH primarily via neurotrophic factor deprivation. However, the specific contributions of individual neuropeptides and their downstream signaling pathways require further mechanistic investigation.

Ferrostatin-1, a ferroptosis inhibitor identified through high-throughput screening of a small molecular library, has been shown to effectively remove alkoxy radicals generated by lipid peroxidation by forming a complex with ferrous ions. This compound exerts a similar anti-ferroptosis effect as GPX4.^49^ Ferrostatin-1 has been demonstrated to mitigate organ damage by inhibiting cell death in lipopolysaccharide-induced acute lung injury and cardiac dysfunction.^50^^,^^51^ In recent years, there has been a growing body of research on the role of ferroptosis in eye diseases. Ferrostatin-1 has been shown to attenuate tissue and cellular damage in diabetic retinopathy by enhancing the antioxidant capacity of the cysteine transport system xc^–^ and GPX4.^52^ Additionally, ferrostatin-1 has been found to reduce retinal denervation induced by light exposure and the development of retinal neovascularization under hypoxic conditions.^53^^,^^54^ In our study, we observed that ferrostatin-1 promoted corneal wound healing, which had been delayed due to corneal TRPV1^+^ sensory denervation. Furthermore, ferrostatin-1 restored corneal sensitivity in mice with TRPV1^+^ sensory denervation, bringing it closer to the level observed in control mice. These findings provide valuable insights into the contributions of TRPV1^+^ sensory nerves and ferroptosis to corneal epithelial homeostasis, offering potential avenues for the development novel therapeutic approaches targeting neurodegenerative diseases and oxidative stress-induced corneal disorders.

AKT/mTOR is an important signal transduction network in cells that plays a crucial regulatory role in physiological activities such as cell proliferation, apoptosis, angiogenesis, and energy metabolism.^55^ It is also closely linked to the initiation and progression of ferroptosis. mTOR signaling is mediated by two distinct complexes, mTORC1 and mTORC2. Notably, mTORC1 inhibition promotes ferroptosis in malignant cells, whereas its activation suppresses ferroptotic cell death.^56^ In the corneal epithelial cells of TRPV1^+^ denervated mice and neurotrophic keratopathy patients, significant upregulation of p53 was observed, which further led to a marked inhibition of the AKT/mTOR signaling pathway. However, it was effectively reversed with the use of ferrostatin-1. This indicates that, in the TRPV1^+^ denervated mice, ferrostatin-1 reactivated the AKT/mTOR signaling pathway by downregulating p53, thereby exerting its biological effects.

Neurotrophic keratopathy is a rare ophthalmic disorder with a prevalence rate below 1:2000.^57^^–^^59^ This rarity presents significant challenges in obtaining a large number of clinical samples, which inherently limited the scope of our study. Despite these limitations, our research provides critical insights into the molecular mechanisms of corneal nerves in regulating corneal epithelial cell responses to ferroptosis. Given the rarity of neurotrophic keratopathy and the challenges in obtaining sufficient clinical samples, our study utilized limited but representative samples to investigate the molecular mechanisms of corneal nerves in regulating corneal epithelial cell responses to ferroptosis. Thus, it is necessary to collect more clinical samples in the future to study new treatment options for this disease. This will not only provide a broader perspective on the clinical implications of our findings but also strengthen the evidence for potential therapeutic interventions.

In conclusion, our study has found that TRPV1^+^ sensory nerve degeneration triggers ferroptosis in corneal epithelial cells, which is reversed by ferrostatin-1 via modulation of the p53/GPX4 canonical pathway. These results offer a promising avenue for advancing therapeutic interventions for neurotrophic keratopathy.

## Supplementary Material

Supplement 1
