# Supplementary material for: Corneal Sensory Denervation Causes Epithelial Ferroptosis and Delayed Healing in Mice
Source: Invest Ophthalmol Vis Sci. 2025 Jun 9;66(6):28. doi: 10.1167/iovs.66.6.28 (PMC12161395; doi:10.1167/iovs.66.6.28)
Supplement: Supplement 1 [file iovs-66-6-28_s001.pdf]

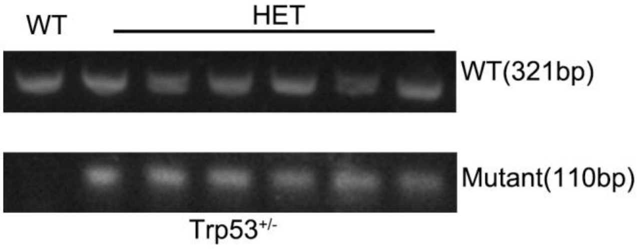

Supplementary Fig. 1. Genotyping of Trp53$^{+/-}$ mice.

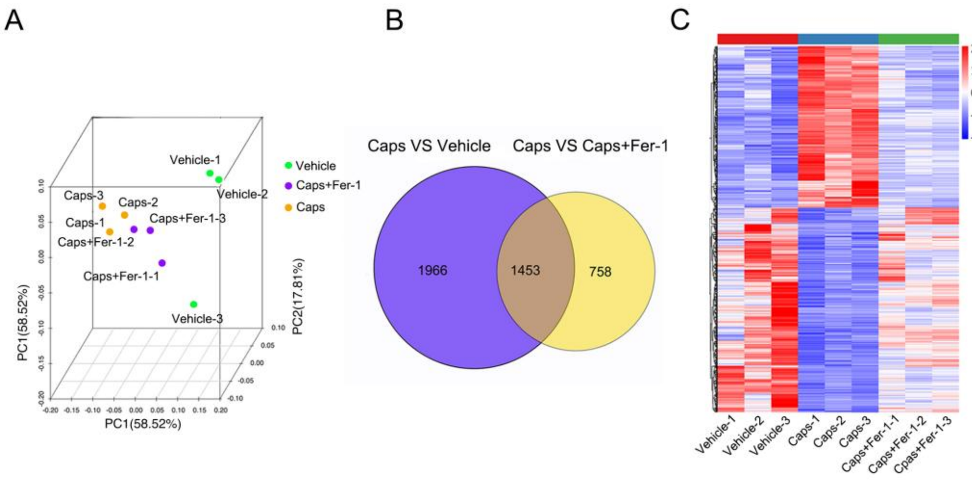

Supplementary Fig. 2. Transcriptome analysis overview of corneal epithelium of capsaicin-treated mice with or without ferrostatin-1 application, and vehicle group. PCA analysis (A), Venn diagram (B) and heat map of 1453 DEGs (C) in the corneal epithelium of capsaicin-treated mice with or without ferrostatin-1 application, and vehicle group.

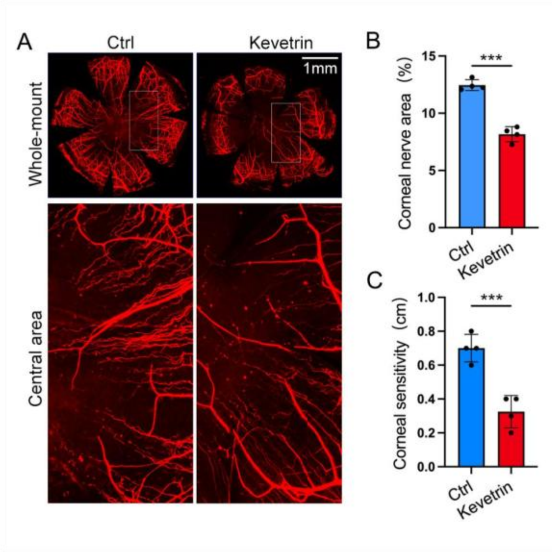

Supplementary Fig. 3. p53 regulates the recovery of corneal nerves during wound healing. (A Corneal nerve regeneration was detected with βIII-tubulin staining at 48 hours after scrape. (B The percentage of nerve-covered area. (C Corneal sensation was measured at 48 hours after scrape.

Supplementary Table 1 Primers used for genotyping of Trp53$^{+/-}$ mice.

Gene	Primers
Trp53	Mutant forward: 5'-CAGCCTCTGTTCCACATACACT-3'
	Wildtype forward: 5'-AGGCTTAGAGGTGCAAGCTG-3'
	Common reverse: 5'-TGGATGGTGGTATACTCAGAGC-3'

Supplementary Table 2 Information of human corneal tissues.

No.	Age	Gender	Diseases Suffered
Nk patient 1	42	female	The patient's right eye developed neurotrophic keratopathy due to brain tumor resection, ultimately leading to corneal perforation
Nk patient 2	56	male	The patient's right eye has developed corneal perforation due to herpetic keratitis
Nk patient 3	56	male	The patient's left eye, which has been repeatedly red and accompanied by a one-year history of decreased vision following an injury, has been diagnosed as viral keratitis
Non-Nk 1	47	male	Enlargement of cerebral hemorrhage hematoma
Non-Nk 2	48	male	Cerebral infarction
Non-Nk 3	40	male	Cerebral hemorrhage

Supplementary Table 3 Primers used for qPCR.

Gene	Primers
β-actin	Forward:5'-ACGGCCAGGTCATCACTATTG-3'
	Reverse:5'-AGAGGTCTTTACGGATGTCAACGT-3'
Gpx4	Forward:5'-GCACATGGTCTGCCTGGATA-3'
	Forward:5'-GGGAAGGCCAGGATTCGTAA-3'
Gstm1	Forward:5'-GAAAGCACCACCTGGATGGA-3'
	Forward:5'-TCCCCAGCAAAGGGTTTG-3'
Gsta2	Forward:5'-GTCCACCTGCTGGAACTTCTT-3'
	Forward:5'-CATCCAAGGGAGGCTTTCTCT-3'
Gsta3	Forward:5'-GGAAGCCAGTCCTTCATTACTTTG-3'
	Forward:5'-GGAACATCAGACTCCCATCACTT-3'
Ccnb1	Forward:5'-TCAAGACTCGACGGGTTGCT-3'
	Reverse:5'-TCAAGACTCGACGGGTTGCT-3'
Trp53	Forward:5'-ACAGCGTGGTGGTACCTTATGA-3'
	Reverse:5'-GGTTCCCACTGGAGTCTTCCA-3'
Lyar	Forward:5'-CGAGGCTCCATCGAAAGGTA-3'
	Reverse:5'-TCCGATGTGTGATGGTCGTT-3'
Slc7a11	Forward:5'-GAGTGCCCGGATCCAGATTT-3'
	Reverse:5'-GGCAACCCCATTAGACTTGTG-3'
